# Physical exercise, fitness and dietary pattern and their relationship with circadian blood pressure pattern, augmentation index and endothelial dysfunction biological markers: EVIDENT study protocol

**DOI:** 10.1186/1471-2458-10-233

**Published:** 2010-05-06

**Authors:** Luis García-Ortiz, José I Recio-Rodríguez, Carlos Martín-Cantera, Alfredo Cabrejas-Sánchez, Amparo Gómez-Arranz, Natividad González-Viejo, Eguskiñe Iturregui-San Nicolás, Maria C Patino-Alonso, Manuel A Gómez-Marcos

**Affiliations:** 1 La Alamedilla Health Centre, Castilla y León Health Service-SACYL, Salamanca, Spain; 2 Passeig de Sant Joan Health Centre. Catalan Health Service-CS. Barcelona. Spain; 3 Cuenca III Health Centre, Castilla La Mancha Health Service-SESCAM, Cuenca, Spain; 4 Casa de Barco Health Centre, Castilla y León Health Service-SACYL, Valladolid, Spain; 5 Torre Ramona Health Centre, Aragón Health Service - Salud, Zaragoza, Spain; 6 Primary Care Research Unit of Bizkaia, Basque Health Service-Osakidetza, Bilbao, Spain; 7 EVIDENT Group. redIAPP: Red de Investigación en Actividades Preventivas y Promoción de la Salud, Spain

## Abstract

**Background:**

Healthy lifestyles may help to delay arterial aging. The purpose of this study is to analyze the relationship of physical activity and dietary pattern to the circadian pattern of blood pressure, central and peripheral blood pressure, pulse wave velocity, carotid intima-media thickness and biological markers of endothelial dysfunction in active and sedentary individuals without arteriosclerotic disease.

**Methods/Design:**

**Design: **A cross-sectional multicenter study with six research groups.

**Subjects: **From subjects of the PEPAF project cohort, in which 1,163 who were sedentary became active, 1,942 were sedentary and 2,346 were active. By stratified random sampling, 1,500 subjects will be included, 250 in each group.

**Primary measurements: **We will evaluate height, weight, abdominal circumference, clinical and ambulatory blood pressure with the Radial Pulse Wave Acquisition Device (BPro), central blood pressure and augmentation index with Pulse Wave Application Software (A-Pulse) and SphymgoCor System Px (Pulse Wave Analysis), pulse wave velocity (PWV) with SphymgoCor System Px (Pulse Wave Velocity), nutritional pattern with a food intake frequency questionnaire, physical activity with the 7-day PAR questionnaire and accelerometer (Actigraph GT3X), physical fitness with the cycle ergometer (PWC-170), carotid intima-media thickness by ultrasound (Micromax), and endothelial dysfunction biological markers (endoglin and osteoprotegerin).

**Discussion:**

Determining that sustained physical activity and the change from sedentary to active as well as a healthy diet improve circadian pattern, arterial elasticity and carotid intima-media thickness may help to propose lifestyle intervention programs. These interventions could improve the cardiovascular risk profile in some parameters not routinely assessed with traditional risk scales. From the results of this study, interventional approaches could be obtained to delay vascular aging that combine physical exercise and diet.

**Trial Registration:**

Clinical Trials.gov Identifier: NCT01083082

## Background

Aging is associated with an increase in arterial stiffness that can be assessed using different tools, such as the increase in pulse pressure, both clinical and ambulatory, changes in circadian pattern of blood pressure, increased pulse wave velocity and central blood pressure, as well as certain biological markers of endothelial dysfunction[1-3].

Blood pressure measurement at the clinic is still currently the standard of reference, but there is increasing evidence that the values obtained after home blood pressure measurement by the patient and particularly 24-hour ambulatory blood pressure monitoring (ABPM) show a better correlation with target organ damage[4] and with cardiovascular events[5,6]. It has been observed that the prognostic value of blood pressure during rest is greater than during activity [7,8] and that a greater variability blood pressure is associated with increased target organ damage[9].

The accepted gold standard to assess arterial stiffness is currently femoral carotid pulse wave velocity (PWV) [1] and it has been related to increased morbidity and mortality in both patients with cardiovascular disease and in healthy subjects[10,11]. It has also been noted that central blood pressure is more strongly related than peripheral blood pressure to cardiovascular morbidity and mortality[12]. The index of increase of central blood pressure, or augmentation index (AIx), is an indicator of central arterial stiffness, which, together with aortic systolic blood pressure and aortic pulse pressure, complements the information obtained with pulse wave velocity to assess arterial stiffness. This index is closely related to both physiological arterial aging and premature aging from certain diseases, though in some studies there are discrepancies in the results obtained[13]. In the ASCOT study [14], increased morbidity and mortality was found in one of the study groups compared to the other, despite the fact that peripheral blood pressures were similar in both groups. In the CAFE sub-study [15], it was found that subjects with greater central blood pressure and AIx had greater morbidity and mortality.

Serum levels of osteoprotegerin (OPG) are elevated in patients with type 1 and 2 diabetes [16,17] from early stages and contribute to the endothelial dysfunction associated with the disease. The OPG has also been found to be associated as a subclinical marker of atherosclerosis in women with postmenopausal osteoporosis and in general population[2,18]. In addition, endoglin levels are decreased in diabetics and are also related to the severity of the vascular involvement[19].

The potential benefits enjoyed by active people, both at the biological and psychological level, range from a better quality of life to a reduction in the risks associated with hypertension, diabetes, cardiovascular diseases and all causes of mortality[20-22]. Some studies have shown a decrease in ambulatory blood pressure with physical exercise[23,24], but the relationship to circadian pattern has not been adequately studied. Aerobic exercise may attenuate arterial hardening as was seen in the Baltimore Longitudinal Study, where elderly male athletes had a PWV, AIx and systolic blood pressure lower than sedentary subjects[25], and it has been associated with a reduced atherosclerosis progression in humans[26]. Improvement of carotid artery elasticity has also been noted in previously sedentary subjects who started a physical exercise program[3]. However, such a clear relationship of arterial stiffness to exercise has not been found in post-menopausal women[27], or in adults with chronic high blood pressure[28]. In addition, it has been observed that high-intensity endurance exercise may be associated with an increase in arterial stiffness[29,30], although this effect could be countered with the performance of aerobic exercise[31].

Therefore, there are still some unresolved issues, such as the effect on vascular structure and function of endurance exercise, alone or combined with aerobic exercise, recommended for maintenance of muscular strength in the elderly[32]. The effect of different doses of exercise on arterial stiffness, circadian pattern of blood pressure, or central blood pressure has not been adequately studied. Finally, in elderly patients with cardiovascular disease, aerobic exercise improved endothelial function[33]. This effect has not been studied or the results are not consistent in healthy subjects[32,34].

There is evidence that the Mediterranean diet (Mediterranean/DASH diet) with high consumption of fruit and vegetables, fish and olive oil may have a positive effect on endothelial function[35-37], as well as on the reduction of peripheral blood pressure[38,39]. However, the effect of dietary patterns on the circadian pattern of blood pressure, particularly on the nocturnal decrease and variability, has not been established, and the influence of these patterns on the biological markers to be analyzed such as OPG and endoglin has also not been clarified. An association has been found between the Mediterranean diet and vascular structure analyzed by carotid intima-media thickness[40]. However, although a decrease in central blood pressure has been found in obese patients subjected to a low-calorie diet[41], the relationship of different dietary patterns with PWV or AIx has not been studied in healthy subjects.

Therefore, the purpose of this study is to analyze the relationship of different doses of physical activity, fitness and dietary pattern to the circadian pattern of blood pressure, vascular structure and function analyzed by PWV and AIx, carotid intima-media thickness and biological markers of endothelial dysfunction (OPG and endoglin). This study continues the research line of the project "Multicenter Assessment of Experimental Program Promoting Physical Activity (PEPAF)" [42,43], focusing on the cardiovascular benefits obtained with healthy lifestyles, taking advantage of the patient cohort of this study and the infrastructure and expertise of the research teams.

### Objectives

1. To analyze the relationship of physical activity and fitness to circadian pattern of blood pressure, central and peripheral blood pressure, PWV, AIx, and carotid intima-media thickness in active and sedentary individuals without arteriosclerotic disease.

2. To analyze the relationship of caloric intake and food pattern to circadian pattern of blood pressure, central and peripheral blood pressure, PWV, AIx and carotid intima-media thickness in active and sedentary individuals without arteriosclerotic disease.

3. To study the correlation of OPG and endoglin levels with arterial stiffness assessed by PWV and AIx, as well as with circadian pattern of blood pressure and carotid intima-media thickness in subjects with different level of physical activity and different nutritional patterns.

## Methods/Design

### Design

A cross-sectional study, to evaluate the association of lifestyles (diet and exercise) with circadian pattern of blood pressure, arterial stiffness and endothelial function in a previously established cohort of healthy subjects with different levels of physical activity. This is a multi-center study with the participation of six groups distributed throughout Spain with the purpose of increasing the sample size to test the proposed hypotheses and to increase the external validity of the study.

### Subjects

#### Study population

Subjects will be selected from the PEPAF project cohort[39,40]. This is a cohort composed of 5,451 subjects identified at the start of the study and divided into three physical activity exposure levels: A) 1,163 sedentary in 2003, who became active in 2006, B) 1,942 who remained sedentary during the 24 months of follow-up, and C) 2,346 who were active at baseline assessment. The study will include subjects aged 20-80 years who agree to participate in this study and sign the informed consent. The exclusion criteria were: known coronary or cerebrovascular atherosclerotic disease, heart failure, moderate or severe COPD, walking-limiting musculoskeletal disease, advanced respiratory, renal or hepatic disease, severe mental diseases, treated oncological disease diagnosed in the past 5 years, pregnant women and terminal patients.

#### Sample size

Sample size was estimated using AIx as a indicator of central arterial stiffness. The CAFE study[15] (ASCOT sub-study[14]) found a difference between the two groups of 6.5 (5.8-7.3) percentage points and this was associated with a difference in cardiovascular morbidity and mortality. The aim is to obtain a size sample sufficient to detect a difference of 3 percentage points of AIx between sedentary, sedentary who became active, and active subjects. There are therefore 3 study groups, assuming a standard deviation of 11, with an alpha risk of 0.05 and a beta risk of 0.20 in a two-sided test, assuming a no response rate of 20%, 352 subjects are required in each group, or a total of 1,056 subjects, but considering a proportional sample to the reference population 1,499 subjects are required. As there will be six participating groups, the sample will be 250 subjects per group for a total of 1,500 subjects, distributed in Groups A, B and C.

#### Sample selection

The sample will be obtained from the subjects remained in the study to the assessment in 2006. Random sampling stratified by groups A, B and C will be performed to obtain a proportional size of subjects in each group for the purpose of obtaining a balanced sample.

### Variables and measurement instruments

#### Anthropometric measurements

Body weight will be determined on two occasions using a homologated electronic scale (Seca 770) following due calibration (precision ± 0.1 kg), with the patient wearing light clothing and no shoes. These readings will be rounded to 100 g. Height in turn will be measured with a portable system (Seca 222), recording the average of two readings, and with the patient shoeless in the standing position. The values will be rounded to the closest centimetre. Body mass index (BMI)(kg/m^2^) will be calculated. Waist circumference will be measured using a flexible graduated measuring tape with the patient in the standing position without clothing. The upper border of the iliac crests are located, and the tape is wrapped around above this point, parallel to the floor, ensuring that it is adjusted without compressing the skin.

#### Office or clinical blood pressure

Office blood pressure measurement involves three measurements of systolic (SBP) and diastolic blood pressure (DBP), using the average of the last two, with a validated OMRON model M7 sphygmomanometer (Omron Health Care, Kyoto, Japan), by following the recommendations of the European Society of Hypertension[44]. Pulse pressure (PP) will be estimated with the mean values of the second and third measurements.

#### Ambulatory blood pressure monitoring

(ABPM): The ABPM will be performed on a day of standard activity, with a radial tonometer. Radial pulse wave acquisition device (BPro) (HealthSTATS Internaciona. 6 New Industrial Road. Singapore 536199. Singapur. Corea) validated according to the protocol of the European Society of Hypertension (ESH), Association for Advancement of medical Instrumentation (AAMI) and British Hypertension society[45] will be used. The records in which the percentage of valid readings was ≥66% of the total and with valid readings at all times were considered to be valid. Furthermore, for the records to be valuable, at least 14 measurements were required during the daytime period, or at least seven during the night time or rest period. The monitor was scheduled for obtaining blood pressure measurements every 15 min during the daytime and rest period. The average and dispersion estimators of SBP and DBP will be calculated during the 24-h, daytime and night time periods, defined based on the diary reported by the patient. The percentage of blood pressure readings above the reference values was also analysed: 24-h blood pressure greater than 130/80 mmHg, daytime blood pressure greater than 135/85 mmHg and night time blood pressure greater than 120 and 70 mmHg for SBP and DBP, respectively[46]. The patients will be classified according to circadian pattern estimated with the nocturnal fall in SBP and DBP. A dipper pattern was defined as a reduction of 10-20%, extreme dipper if reduction was higher than 20%, non dipper if it was 0-10% and riser if it was below 0% for both SBP and DBP. The patients will be classified too by SBP nigh/day ratio and in Dipper < 0,9, Non Dipper between 0,9 - 1, and Risser > 1.

#### Central blood pressure and augmentation index

Central blood pressure will be measured with Pulse Wave Application Software (A-Pulse) (HealthSTATS International, 6 New Industrial Road, Singapore 536199. Singapore, Korea) using tonometry to capture the radial pulse and by equation patented estimate central blood pressure. The increase in central blood pressure, mean blood pressure and radial Augmentation index will be estimated. Radial AI will be calculated as follows: (Second peak systolic blood pressure [SBP2] - diastolic blood pressure [DBP])/(first peak SBP - DBP) × 100 (%).

It is the same instrument that performs the 24 ABPM (BPro), with an specific software to determine the central blood pressure and estimation of the parameters derived from it.

#### Pulse wave analysis (PWA) and pulse wave velocity (PWV)

In the Salamanca cohort, AIx and PWV will also be estimated using the SphymgoCor System (AtCor Medical Pty Ltd Head Office, West Ryde, Australia), currently the gold standard for measuring arterial stiffness[1], and will serve as a quality control for the measurements obtained with the Pulse Wave Application Software (A-Pulse). Using the SphygmoCor System (Px Pulse Wave Analysis), with the patient in the sitting position and resting the arm on a rigid surface, pulse wave analysis will be made with a sensor in the radial artery, using mathematical transformation to estimate the aortic pulse wave. From the morphology of the aortic wave, central (aortic) blood pressure, central ventricular load, diastolic perfusion pressure, subendocardial viability index, the pressure increase, central pressure pulse and Central AIx will be estimated. Central AIx, will be estimated using the following formula: Increase in central pressure * 100/Pulse pressure. Using the SphygmoCor System (Vx Pulse Wave Velocity), and with the patient in the supine position, the pulse wave of the carotid and femoral arteries will be analyzed, estimating the delay with respect to the ECG wave and calculating the PWV. Distance measurements will be taken with a measuring tape from the sternal notch to the carotid and femoral arteries at the sensor location.

#### Assessment of carotid intima-media thickness

Carotid ultrasonography to assess IMT will be performed by two investigators specifically trained for this before starting the study. A Sonosite Micromax ultrasound (*Sonosite Inc., Bothell, Washington, USA*) device paired with a 5-10 Mhz multifrequency high-resolution linear transducer with Sonocal software will be for performing automatic measurements of IMT for optimising reproducibility. Measurements will be made of the primitive carotid after the examination of a longitudinal section of 10 mm at a distance of 1 cm from the bifurcation, performing measurements in the anterior or proximal wall, and in the posterior or distal wall in the lateral, anterior and posterior projections, following an axis perpendicular to the artery to discriminate two lines, one for the intima-blood interface and the other to the media-adventitious interface. A total of 6 measurements will be obtained of the right carotid and other 6 of the left carotid, using average values (average IMT) and maximum values (maximum IMT) calculated by the software automatically. The measurements will be obtained with the subject lying down, with the head extended and slightly turned opposite to the carotid examined, following the recommendations of the Manheim Carotid Intima-Media Thickness Consensus[47]. Finally, IMT image is frozen in telediastole by means of electrocardiogram triggering to avoid a confounding effect of pulsatile deformation of wall thickness and transferred to a computer. The average IMT will be considered to be abnormal if it is above 0.9 mm, or if there is atherosclerotic plaques with a diameter over 1.5 mm, or a focal increase of 0.5 mm, or 50% of the adjacent IMT [47].

#### Evaluation of peripheral artery involvement

This will be evaluated using the ankle-brachial index (ABI), performed in the morning without having consumed coffee or tobacco for at least 8 hours prior to measuring and an ambient temperature of 22-24°C. With the feet uncovered, in a supine decubitus position after 20 minutes of rest, the pressure in the lower extremities was measured using a portable Doppler system Minidop Es-100Vx (*Hadeco, Inc. Arima, Miyamae-ku, Kawasaki, Japan*) applying the probe at the anterior or posterior tibial artery at an angle of approximately 60° to the direction of blood flow. The transducer's cuff will be quickly inflated in each ankle about 30 mmHg above the systolic pressure and the pressure will be allowed to descend (by about 2 mmHg per second) until the first sound corresponding to the systolic pressure was heard. The blood pressure will be also measured in both arms (measured twice at 3-5 minute intervals). The ABI will be calculated separately for each foot by dividing the higher of the two systolic pressures in the ankle by the highest measurement of the two systolic pressures in the arm [48].

#### Physical activity

Physical activity will be estimated by the 7-day Physical Activity Recall (PAR) and accelerometer. The PAR is a general measure of physical activity, which has been recognized as valid and reliable tool in recent years and is widely used in epidemiological, clinical and behavior change studies. It consists of a semi-structured interview (10-15 minutes) in which participants provide an estimate of the number of hours dedicated to physical or occupational activities requiring at least a moderate effort in the past seven days. The categories collected are: moderate, vigorous, and very vigorous physical activity. The amount of time dedicated to each activity is multiplied by the mean metabolic equivalents (METs) of each category: light activity 1.5, moderate 4, vigorous 6, and very vigorous 10. The sum of the products of the hours dedicated to each activity and its estimated mean energy expenditure (MET) provides an estimation of the kilocalories per kilogram used per day (kcal*kg-1 * d-1). The dose of physical exercise will be estimated in METs/hour/week and active persons were considered as those doing at least 30 minutes of moderate activity, five days a week, or at least 20 minutes of vigorous activity, 3 days a week. Persons not reaching this level of physical activity were considered sedentary[49].

#### Accelerometer

Actigraph GT3X accelerometers (Actigraph, Shalimar, FL, USA) will be used, which have been previously validated[50]. Subjects will wear the accelerometer fastened with an elastic strap to the right side of waist for seven consecutive days, except for bathing and performing activities in the water. The data will be recorded at 1-minute intervals. Total physical activity will be expressed in counts per minute. The intensity of physical activity (low, moderate or high) will be determined according to the cut-off points proposed by Freedson[51].

#### Physical fitness

Measurement of physical fitness will focus on estimating cardiorespiratory fitness, defined as maximal oxygen consumption (VO_2 _max) and physical work capacity using a submaximal test. Evaluation of physical fitness or maximal oxygen consumption will be made using indirect exercise tests (which are simpler and safer), extrapolating the data obtained from the work load performed (watts) and heart rate using the PWC-170 or physical work capacity at 170 beats per minute.

Assessment of usual dietary intake: The food intake frequency questionnaire of the University of Navarra validated for Spain will be used[52]. This questionnaire will be self-administered following patient training by a nurse. The questionnaire asks about the frequency of intake of 137 standard foods in the reference population. This estimated frequency corresponds to the previous year at the time of the interview and is divided into 9 intake frequencies ranging from never to more than 6 times daily. This will be used to estimate daily energy intake, essential nutrients, sodium, fiber, folic acid, iron, calcium, antioxidants (vitamin C, E, selenium), and other nutrients.

#### Other variables

age, sex, occupation, tobacco and alcohol consumption, family and personal history of risk factors (arterial hypertension, dyslipidemia and diabetes) and cardiovascular diseases and drug use. A blood sampling will be drawn to assess lipids, blood glucose, HbA1c, blood insulin, and renal function, and a urine sample will be collected to assess index albumin/creatinine. A blood sample will be frozen for subsequent determination in the laboratory of the physiology department of the University of Salamanca of biological markers of endothelial dysfunction, OPG and endoglin in plasma by ELISA using specific commercial kits and following the manufacturer's instructions.

### Statistical analysis

Data input will be made using the Teleform system (Autonomy Cardiff Vista, California, USA), with a questionnaire previously designed for the project. Results will be expressed as mean ± standard deviation for quantitative variables or by frequency distribution for qualitative variables. The Pearson chi-square test will be used to analyze associations between qualitative variables. Student's t test for independent samples will be used to compare the means of the two groups and an ANOVA will be performed if the number of groups is increased. Post hoc comparisons will then be performed using the LSD method, with alpha < 0.05. The relationship between quantitative variables will be analyzed using Pearson's correlation coefficient. Finally, a multivariate analysis of multiple linear regression and logistic regression will be made to analyze the most significant variables (physical activity, physical fitness and dietary pattern) circadian pattern, arterial stiffness (PWV and AIx), and endothelial dysfunction (endoglin and OPG). Physical exercise will be analyzed to determine changes in cardiovascular parameters according to the dose of physical exercise performed. For hypothesis testing, an alpha risk of 0.05 will be set as the limit of statistical significance. SPSS/PC+ version 15.0 statistical package will be used (SPSS Inc., Chicago, Illinois, USA).

### Quality control

In order to ensure data quality, the nursing professionals in charge of data collection will receive specific training. Regular external monitoring will then be performed in the six health centers to verify adequate application of methods, both in performing the different examinations and collecting the information. At La Alamedilla health center, a measurement of arterial stiffness (PWV and AIx) with SphygmoCor System (gold standard) for the purpose of confirming correct determination of these parameters.

### Methodological limitations

The main limitation in the first phase of the study is that it is a cross-sectional design and therefore only allows detection of association and not causal relationship. This problem is expected to be solved in the second phase with randomized clinical trial that is planned. Selection of the sample was randomized and so it would ideally represent the population without cardiovascular disease seen in primary care, but the losses suffered by the cohort during follow-up were close to 20% and this may have influenced the representativeness of the population attending primary care centers.

### Ethical and legal issues

In order to guarantee data confidentiality, all the electronic and paper copies of the protocol, signed informed consent documents and results of the tests made in each of the patients will be kept locked in a safe place, and only the study investigators will have access to the data on the subjects who agree to participate in the study.

The study has been approved (April 24, 2009) by the research ethics committee from health area of Salamanca and complies with Spanish data protection law 15/1999 and its recently developed specifications (Royal Decree (RD) 1720/2007). Knowledge and agreement to cooperate has been established with the implicated services, signed by the legal representative of the centre.

## Discussion

It is expected to confirm the hypothesis that healthy lifestyles -physical activity and healthy diet- produce an improvement in circadian pattern of blood pressure, PWV, and AIx as indicators of central arterial stiffness and impaired vascular function, in carotid intima-media thickness as an indicator of impaired vascular structure, and in endothelial damage assessed by endoglin and OPG.

Determining that sustained physical activity and the change from sedentary to active as well as a healthy diet improve circadian pattern, arterial elasticity and carotid intima-media thickness may improve lifestyle intervention programs. These interventions could improve cardiovascular risk profile in some parameters not routinely assessed with the traditional risk scales. This baseline assessment will provide essential information for the subsequently planned interventional clinical trial to test the improvement in these parameters with a specific intervention.

The use of a new tool such as the Radial Pulse Wave Acquisition Device (BPro) and Pulse Wave Application Software (A-Pulse), and verification of their feasibility and convenience of use may help to generalize the evaluation of certain cardiovascular parameters that increase vascular risk and are not routinely assessed. If our hypotheses are confirmed, molecules such as OPG or endoglin could be used as early markers of endothelial dysfunction.

From the results of this study, an interventional approach could be obtained to delay arterial aging that combines physical exercise and diet. This approach based on healthy lifestyles could be a powerful tool to stop vascular aging.

## Abbreviations

ABPM: Ambulatory blood pressure monitoring; PWV: Pulse Wave Velocity; AIx: Augmentation Index; ASCOT: Anglo-Scandinavian Cardiac Outcomes Trial; CAFÉ: Conduit Artery Function Evaluation; OPG: Osteoprotegerina; DASH: Dietary Approaches to Stop Hypertension; PEPAF: Experimental Program for Physical Activity Promotion; BMI: Body mass index; SBP: Systolic blood pressure; DPB: Diastolic blood pressure; ESH: European Society of Hypertension; AAMI: Association for Advancement of medical Instrumentation; PWA: Pulse Wave Analysis; IMT: Intima-media thickness; ABI: Ankle-brachial index; PAR: Physical Activity Recall; MET: Metabolic equivalent; VO2max: Maximum oxygen consumption.

## Competing interests

The authors declare that they have no competing interests.

## Authors' contributions

Conception of the idea for the study: LGO, MAGM and JIRR. Development of the protocol, organization and funding: LGO, JIRR, C MC, ACS, AGA, NGV, EISN, CPA and MAGM. Writing of the manuscript: LGO and MAGM. All the authors have read the draft critically, to make contributions, and have approved the final text. The project will be developed by Evident group.

## Pre-publication history

The pre-publication history for this paper can be accessed here:

http://www.biomedcentral.com/1471-2458/10/233/prepub
